# Regulatory B Cells at the Crossroads of Epigenetic Control and Immune Homeostasis

**DOI:** 10.1007/s12016-026-09140-y

**Published:** 2026-04-08

**Authors:** Duminduni Hewa Angappulige, Darby J. Ballard, Christina James Thomas, Shan Xu, Xinlei Guo, Yashvi Bhavin Gandhi, Sushim Mishra, Paul de Figueiredo, Jianxun Song

**Affiliations:** 1 Department of Microbial Pathogenesis and Immunology, Texas A&M University Health Science Center, MREB II, Room 3344, 8447 John Sharp Pkwy, Bryan, TX 77807 USA; 2 Department of Veterinary Medicine and Biomedical Sciences, Texas A&M University, College Station, TX 77843 USA; 3 Department of Nutrition, Texas A&M University, College Station, TX 77843 USA; 4 Bond Life Sciences Center, The University of Missouri, 1201 Rollins Street, Rm 240, Columbia, MO 65211 USA

**Keywords:** Regulatory B cells (Bregs), Regulatory T cells (Tregs), Immunosuppression, Autoimmunity, Cancer, Interleukin-10 (IL-10), Histone deacetylases (HDACs), Epigenetic regulation, Blimp-1, STAT3, c-Maf

## Abstract

Regulatory B cells (Bregs) cooperate with FOXP3⁺ regulatory T cells (Tregs) to maintain immune tolerance. Among diverse Breg subsets, IL-10⁺ Bregs are the best-defined mediators of suppression across autoimmunity and cancer. We synthesize evidence that histone deacetylases (HDACs) are the central epigenetic switch coupling cytokine and checkpoint signals to the Il10 locus, thereby programming Breg identity and stabilizing immunosuppression. Mechanistically, Blimp-1, STAT3, and c-Maf integrate environmental cues (e.g., LPS, IL-21, TIM-1 ligation) with HDAC-directed chromatin accessibility, while BACH2 and HDAC3 restrain Prdm1 to gate plasmablast-like Breg differentiation. We outline how this HDAC–IL-10 module coordinates Breg–Treg crosstalk (CD40/CD40L, PD-1/PD-L1, TIGIT, adenosinergic CD39/CD73) to suppress Th1/Th17 effectors. Disease context dictates outcome: Breg deficits fuel autoimmunity, whereas Breg expansion/reprogramming in tumors dampens cytotoxic immunity (PD-L1⁺, VISTA⁺, IL-35⁺ Bregs). We propose a framework for precision epigenetic modulation: enhancing HDAC-sensitive Breg programs to restore tolerance in autoimmunity, and disrupting tumor-skewed Breg circuits (PD-L1/VISTA, IL-35/STAT3, adenosine metabolism) to improve cancer immunotherapy. This perspective unifies Breg heterogeneity under a tractable axis—HDAC-tuned IL-10 chromatin—and highlights clinically actionable levers at the interface of epigenetics and immune regulation.

## Highlights

IL-10-producing Bregs act in concert with Tregs to maintain immune tolerance during inflammation/promote immunosuppression during inflammation.

Histone deacetylases (HDACs) tune IL-10 chromatin to set Breg state; Breg–Treg crosstalk then enforces tissue-specific tolerance or tumor immune escape.

Epigenetically regulate IL-10 expression in both Bregs and Tregs.

The transcriptional regulators Blimp-1, STAT3, and c-Maf integrate cytokine signaling with epigenetic control of IL-10-driven immunosuppression.

## Background

The vertebrate immune system faces the dual challenge of mounting robust defenses against pathogens while avoiding excessive inflammation and autoimmunity. Specialized subsets of regulatory immune cells have evolved to maintain this delicate equilibrium. Regulatory T cells (Tregs), defined by expression of the transcription factor FOXP3, and regulatory B cells (Bregs), a functionally defined subset of B lymphocytes, act in concert to enforce immune tolerance [1, 2].

Central to their function is the induction and maintenance of self-tolerance. During lymphocyte development in the thymus and bone marrow, clonal deletion and anergy eliminate autoreactive clones, establishing a baseline of central tolerance [3, 4]. However, this process is incomplete, and autoreactive cells can often escape into the periphery. In this context, Tregs and Bregs are essential guardians of peripheral tolerance, suppressing pathogenic responses against self-antigens while allowing effective immunity against foreign threats [5].

Bregs and Tregs function through overlapping yet distinct mechanisms. Tregs primarily exert suppression through cell-cell contact and the secretion of cytokines such as interleukin-10 (IL-10) and transforming growth factor-beta (TGF-β) [6]. To sustain their anti-inflammatory role, Bregs similarly secrete IL-10, along with interleukin-35 (IL-35) or TGF-β, which maintains their suppressive capacity [7, 8]. Together, FOXP3^+^ Tregs and IL-10^+^ Bregs form a layered immunoregulatory network that prevents uncontrolled inflammation.

Emerging evidence underscores the importance of epigenetic regulation in stabilizing the suppressive functions of these regulatory cells. Histone deacetylases (HDACs) suppress IL-10 expression in Bregs and increase histone acetylation [9]. This indicates that HDAC11 binds to the Il-10 promoter, limiting chromatin accessibility at the IL-10 locus. Inhibition of HDACs reverses this repression, facilitating transcription factor recruitment and restoring the IL-10-suppressive phenotype of Tregs [10]. Since HDAC inhibition in Tregs reflects a broader mechanism of Breg programming and highlights opportunities to manipulate epigenetic regulation, this could be used therapeutically to enhance immunosuppression in autoimmune conditions and to counter immune evasion in cancer. This review explores the phenotypic and functional diversity of Bregs in mice and humans, examines the molecular and epigenetic mechanisms that govern their suppressive activity, and discusses the translational implications of targeting the HDAC-IL-10 axis in autoimmunity and cancer.

## Regulatory B Cells: an Overview

B lymphocytes are classically known for their roles in antibody secretion, functioning as antigen-presenting cells (APCs), and the generation of immunological memory. However, subsets of B cells also acquire potent immunoregulatory functions. These regulatory B cells, collectively termed Bregs, suppress pathogenic T cell responses and promote immune tolerance. Unlike Tregs, which are defined by lineage commitment to FOXP3, Bregs are identified primarily by function. Their phenotypic heterogeneity and the absence of a single lineage-defined transcription factor complicate their classification.

**Fig. 1:**
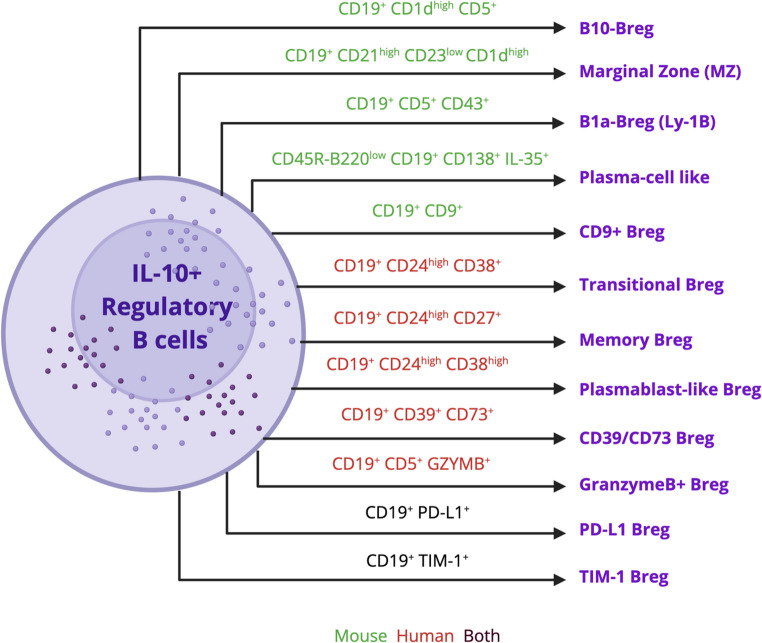
Phenotypic identification of IL-10 + producing regulatory B cells (Bregs). Different phenotypic markers characterize Bregs in mice and humans

Instead, Bregs are distinguished by their ability to secrete immunosuppressive cytokines, particularly IL-10, IL-35, and TGF-β, and their capacity to suppress effector T cell proliferation and inflammatory cytokine production. The best-characterized Breg subsets include IL-10-producing B10 cells (CD19^+^CD5^+^CD1d^hi^), transitional Bregs (CD19^+^CD24^hi^CD38^+^), memory Bregs (CD19^+^CD24^hi^CD27^+^), plasmablast-like Bregs, marginal zone (MZ) Bregs, TIM-1^+^ Bregs, PD-L1^+^ Bregs, and Granzyme B^+^ Bregs (Fig. 1; Table 1). Each of these subsets exhibits distinct surface markers and functional specializations, yet all converge on the shared goal of immune suppression.

Furthermore, Bregs are found in both lymphoid and peripheral tissues, with their proportions changing according to the tissue microenvironment’s physiological condition. The CD19^+^CD24^hi^CD38^hi^ regulatory B cell population was examined in healthy umbilical cord blood (hUCB) and healthy adult peripheral blood (hAPB) [11]. Compared with total regulatory B cells in blood, hUCB had a higher proportion of Bregs (34.39%), while hAPB showed a lower proportion (9.49%). Zhao and Jung [12] demonstrated that both peripheral blood (PB) and synovial fluid (SF) from patients with juvenile idiopathic arthritis (JIA) patients showed reduced percentages of CD19^+^CD24^hi^CD38^hi^ regulatory B cells compared to controls. Specifically, Breg% in PB was 16.11% compared to 23.83% in controls, and in SF it was 3.23% compared to 16.11% in controls [12]. The proportion of Bregs (CD19^+^CD24^hi^CD38^hi^) in the peripheral blood of dermatomyositis (DM) patients was reduced to 0.713%, compared to 4.196% in healthy controls [13]. Similarly, IL-10^+^ B cells were decreased in the blood of DM patients (1.139%) compared to healthy controls (2.372%) [13]. Additionally, 1–2% of splenic CD1d^hi^ CD5^+^ Bregs, which are essential for suppressing Th1-driven inflammation [14]. Furthermore, the IL-10-producing B10 Bregs (CD5^+^ B-1a cells) in the peritoneal cavity helps reduce colitis-mediated inflammation, thereby supporting the gastrointestinal tissue homeostasis. These peritoneal cavity B cells are 10-fold higher than those in mesenteric lymph nodes [15].

### Phenotypic Identification of Mouse Bregs

#### B10 Cells

Murine systems have been instrumental in defining the functional and phenotypic diversity of Bregs. Among these, B10 cells represent the prototypical IL-10-producing Bregs. First described as a distinct regulatory subset, B10 cells are identified by the CD19^+^CD1d^hi^CD5^+^ phenotype and enriched within the spleen. Functionally, they exert potent suppression of T cell-mediated inflammation through IL-10 secretion, and their depletion of dysfunction leads to exacerbated autoimmune pathology [14]. Consistent with their suppressive role, CD1d-expressing Bregs have also been implicated in the regulation of gut-associated inflammatory responses, highlighting their importance at the interface of systemic and mucosal immunity [7].

#### Marginal Zone (MZ) and B1a Bregs

Beyond B10 cells, several additional subsets of murine Bregs have been defined based on surface markers, anatomical location, and functional activity. Marginal zone (MZ) Bregs, characterized by CD19^+^CD21^hi^CD23^lo^CD1d^hi^, reside in the splenic marginal zone and play an essential role in regulating systemic inflammation [16]. B1a Bregs, identified as CD19^+^CD5^+^CD43^+^, originate in the peritoneal cavity and contribute to IL-10-dependent suppression of effector T cell (Teffs) responses, particularly in the tumor microenvironment (TME) [17]. These subsets emphasize the anatomical specialization of Bregs in murine systems, with distinct compartments contributing to tissue-specific immunoregulation.

#### CD9^+^ Bregs

Transcriptomic and functional profiling have further revealed a CD9^+^ Breg population that spans MZ, B1a, and B1b compartments. CD9^+^ Bregs have robust immunosuppressive activity, potently inhibiting T cell proliferation and amplifying anti-inflammatory responses. Their relevance has been defined in models of allergic airway inflammation, where expansion of CD9^+^ Bregs correlates with reduced inflammatory pathology [18, 19]. Murine Bregs cannot be confined to a single developmental lineage, but rather represent a functional program inducible to multiple B cell states.

#### TIM-1^+^ Bregs

TIM-1^+^ Bregs provide a compelling example of receptor-driven regulatory specialization. In murine islet transplantation models, TIM-1^+^ Bregs promote graft tolerance by suppressing Th1 and Th17 effector responses in an IL-10-dependent manner [20]. TIM-1 deficiency (TIM-1^−/−^) impairs apoptotic cell recognition, reduces IL-10 production, and culminates in multi-organ inflammation, demonstrating the non-redundant role of TIM-1 in stabilizing Breg identity and function [21]. Checkpoint-like molecules directly shape the regulatory phenotype of murine B cells.

#### Plasmablast-like IL-35^+^ Bregs

Additional murine subsets extend the functional breadth of Breg biology. IL-35^+^ plasmablast-like cells (CD19^+^CD138^+^) have been identified as protective in experimental autoimmune encephalomyelitis (EAE), where they constrain pathogenic T cell responses and ameliorate disease severity. Importantly, IL-35^+^ and IL-10^+^ plasma cells appear to constitute functionally distinct Breg populations, coexisting within the IgM^+^CD138^hi^TACI^+^CXCR4^+^CD1d^int^TIM-1^int^ plasma cell pool, as revealed in the context of *Salmonella* infection [22]. Similarly, PD-L1^+^ Bregs have been shown to regulate adaptive immune responses by suppressing T follicular helper (Tfh) cells expansion and restraining T cell differentiation, particularly in the EAE model [23].

Collectively, these studies demonstrate that murine Bregs encompass a diverse repertoire of subsets defined by both surface phenotype and functional specialization (Fig. 1; Table 1). The abundance and activity of these populations are highly context-dependent, shaped by local cues from infection, autoimmunity, transplantation, or the TME. Murine models reveal that Bregs represent a flexible, inducible regulatory program that can be superimposed upon multiple B cell developmental lineages, providing a dynamic means of restraining inflammation and maintaining immune tolerance.

### Phenotypic Identification of Human Bregs

#### Transitional Bregs

In humans, immature B cells arising in the bone marrow enter a transitional phase during which they circulate through peripheral blood before homing to secondary lymphoid tissues such as the spleen for maturation. Within this developmental window, a subset acquires immunoregulatory function and is commonly referred to as transitional Bregs. Phenotypically, transitional Bregs are enriched within the CD19^+^CD24^hi^CD38^hi^ compartment and exhibit potent suppressive activity. Clinically, their relevance is spotlighted by observations in systemic lupus erythematosus (SLE), where transitional Bregs are numerically reduced and functionally impaired, coinciding with heightened Th1 polarization and a diminished capacity to restrain T cell responses [24]. Consistent with this, transitional Bregs effectively suppress pro-inflammatory cytokine production by CD4^+^ T cells, positioning them as frontline regulators in the peripheral blood and spleen [25].

#### Memory Bregs

Beyond the transitional compartment, memory Bregs constitute another major human regulatory subset and are typically defined as CD19^+^CD24^hi^CD27^+^ cells. These memory Bregs contribute to durable immune tolerance, in part by sustaining IL-10 production and limiting pathogenic Th1 responses. Their clinical significance is accentuated in Graves’ disease (GD), where peripheral blood memory Bregs display a reduced frequency and impaired IL-10 output, correlating with elevated IFN-γ production by CD4^+^ T cells and disease activity [26]. Together with data showing that human memory B cells can give rise to B10-like IL-10-producing subsets, these findings support a model in which memory Bregs provide long-term regulatory functionality and help maintain peripheral tolerance [25].

#### Plasmablast-like Bregs

Plasmablast-like Bregs (CD27^+^CD38^hi^) represent a functionally distinct arm of the human Breg continuum. Although plasmablasts are classically viewed as antibody-secreting precursors, a subset identified within the CD27^+^CD38^hi^ gate can produce substantial IL-10 and exert immunoregulatory effects. Notably, this regulatory program is context-dependent: IL-10-producing plasmablast-like Bregs lose suppressive function in chronic graft-versus-host disease (cGVHD), illustrating how inflammatory milieus can reshape B-cell regulatory capacity [27]. Additional evidence of environmental sensitivity comes from sepsis, where absolute numbers of CD19^+^CD24^hi^CD38^hi^ transitional B cells are reduced in peripheral blood relative to healthy controls, a change that likely contributes to the dysregulated immune set point characteristic of severe systemic inflammation [28].

Single-cell mass cytometry and transcriptional profiling have further expanded the human Breg landscape by resolving proliferative, functionally suppressive clusters that do not neatly conform to classical gates. In non-small-cell lung cancer (NSLSC), a plasmablast-like Breg population characterized by Ki-67^+^CD27^hi^CD38^hi^CD95^hi^ and intermediate IL-10 expression has been described, indicating that cycling B cells within the tumor microenvironment (TME) can adopt regulatory phenotypes [29]. The same study identified a punitive B10-like cluster (cluster 19: CD5^hi^CD25^hi^ CD24^+^PD-1^+^ IL-10^lo^) with suppressor potential, as well as immature PD-L1^+^IL-10^+^ Bregs consistent with checkpoint-mediated regulation [29]. These data enforce an emerging principle: human Bregs are not a single static subset, but a family of states whose frequency, phenotype, and suppressive potency are molded by inflammatory cues and tissue context.

#### CD39^+^/CD73^+^ and TIGIT^+^ Memory Bregs

Adenosinergic Bregs add a complementary, contact-independent layer of suppression. Co-expression of CD39 and CD73 on IL-10-producing B cells identifies a CD19^+^CD39^+^CD73^+^ subset capable of converting extracellular ATP to adenosine (ADO), thereby dampening inflammation and supporting immune homeostasis across autoimmune and inflammatory settings [30, 31]. In SLE, frequencies of CD39^+^/CD73^+^ Bregs are reduced and serum ADO levels are diminished, suggesting that defects in purinergic metabolism compound the loss of IL-10-mediated control and contribute to disease flares [32]. Checkpoint-linked memory Bregs further illustrate the integration of metabolic and inhibitory pathways in human tolerance. TIGIT^+^ memory B cells that co-express CD39^+^/CD73^+^ (CD19^+^CD24^hi^CD27^+^CD39^hi^IgD^−^IgM^+^CD1c^+^) have been implicated in maintaining allograft tolerance, likely by curbing Tfh cell activity and restraining alloantibody generation. Clinically, donor-specific antibody (DSA)-positive transplant recipients show decreased TIGIT^+^CD39^+^/CD73^+^ Bregs, which are associated with heightened inflammation, impaired regulation of Tfh cells, and graft dysfunction [33]. This suggests that preserving or restoring TIGIT^+^ adenosinergic Bregs could be an actionable strategy to stabilize humoral tolerance after transplantation.

#### TIM-1^+^ Bregs

TIM-1-expressing Bregs constitute another checkpoint-associated regulatory axis with divergent roles in cancer and autoimmunity. In hepatitis B-virus-related hepatocellular carcinoma, TIM-1^+^ Bregs produce IL-10 and inversely correlate with cytotoxic CD4^+^ T cell activity (Granzyme A^+^Granzyme B^+^perforin^+^), indicative of compromised anti-tumor immunity in the TME [34]. Tumor-associated TIM-1^+^ Bregs also accumulate in human cutaneous squamous cell carcinoma (SCC), consistent with a broader role for TIM-1-dependent B cell regulation in facilitating immune evasion [35]. In contrast, patients with autoimmune disorders such as systemic sclerosis exhibit decreased frequencies and functional impairment of TIM-1^+^ Bregs, aligning with the concept that insufficient B-cell regulation can permit unchecked inflammation and tissue damage [36]. The bidirectional association of TIM-1^+^ Bregs with disease, protective in autoimmunity when present, permissive of tumor growth when enriched, stresses the context-specific implications of Breg biology.

#### PD-L1^+^ Bregs

PD-L1^+^ Bregs likewise display disease-specific behavior. In melanoma, higher frequencies of PD-L1^+^ Bregs correlate with advanced clinical stage and the capacity to suppress T cell responses, including bone metastases [37]. By contrast, PD-L1^+^ B cells derived from the peripheral blood of rheumatoid arthritis (RA) patients fail to suppress IFN-γ and IL-21 production, suggesting qualitative defects in checkpoint-mediated regulation that may exacerbate autoantibody production and joint inflammation [38]. These observations argue that PD-L1 expression on B cells is not uniformly predictive of suppression; rather, its functional impact depends on the co-inputs from cytokines, tissue-resident signals, and the differentiation state of the B cell.

#### Granzyme B^+^ Bregs

Cytotoxic-programmed Bregs induced by IL-21 add an additional, less appreciated dimension to human B cell regulation. Granzyme B-producing CD5^+^ B cells can directly inhibit T cell proliferation, extending the toolkit of B cell-mediated suppression beyond IL-10 and adenosine pathways [39]. However, as with other Breg subsets in autoimmune settings, GrB^+^ Bregs possess functional impairments in RA, particularly in the face of Th1 and Th17 skewing, reinforcing the theme that inflammatory cytokine milieus can erode B cell regulatory programs [40].

Human Bregs comprise a phenotypically diverse and highly plastic network that spans transitional, memory, plasmablast-like, adenosinergic, and checkpoint-linked states (Fig. 1; Table 1). Their defining function, suppression of effector T cells, modulation of Tfh activity, and reinforcement of peripheral tolerance, are achieved through convergent mechanisms. These mechanisms include IL-10 production, purinergic signaling via CD39/CD73, checkpoint engagement through PD-L1 and TIGIT, and, in specific contexts, cytotoxic programs driven by IL-21. Disease-specific perturbations across these axes reveal a consistent pattern. In autoimmunity, quantitative and qualitative deficits in Bregs undermine tolerance, whereas in cancer, the expansion or reprogramming of Bregs in the TME can dampen anti-tumor immunity. This duality emphasizes why precise phenotyping, integrating classical surface markers with functional readouts and single-cell states, remains essential for translating Breg biology into target interventions.

**Table 1:** Phenotypic identification of regulatory B cells

Breg Subset	Species	Phenotypic Markers	Functional Role	Cytokines Secreted
B10	Mouse	CD19^+^ CD1d^hi^ CD5^+^	● Inhibits disease severity in intestinal inflammation/colitis model [41] ● Suppresses T-cell-mediated inflammation in the EAE mouse model of MS	IL-10
Marginal Zone (MZ)	Mouse	CD19^+^ CD21^hi^ CD23^lo^ CD1d^hi^	● Regulates intestinal inflammation in colitis model [7] ● Promotes IL-10-mediate immunosuppression	IL-10
B1a-Bregs (Ly-1B cells)	Mouse	CD19^+^ CD5^+^ CD43^+^	● Promotes the inhibition of macrophage activation upon LPS stimulation ● Suppresses Th1-mediated immune response [42] ● Promotes melanoma tumor growth ● Suppresses Th1-mediated immune response in CD8 + cells [17]	IL-10
Plasmablast/ Plasma cell-like	Mouse	CD45R(B220)^lo^ CD19^+^ CD138(Syndecan-1)^+hi^ IL-35^+^	● Suppresses EAU by reducing Th1/Th17 [8] ● IL-35^+^ Bregs suppress EAE [22]	IL-10 IL-35
CD9^+^	Mouse	CD19^+^ CD9^+^	● Induces T cell apoptosis [19] ● Reduces lung inflammation in vivo [18]	IL-10
Transitional	Human	CD19^+^ CD24^hi^ CD38^+^	● Suppresses Th1/Th17 [25] ● Dysfunctional during autoimmune condition (SLE) [24]	IL-10 TGF-β
Memory	Human	CD19^+^ CD24^hi^ CD27^+^	● Inhibits CD4^+^ T cell proliferation [25]	IL-10
Plasmablast-like	Human	CD27^hi^ CD38^hi^ CD95^hi^ IL-10^int^	● Decreases anti-tumor immunity in NSCLC [29] ● Regulates autoimmunity and immune tolerance	IL-10 IL-35
CD39/CD73	Human	CD19^+^ CD39^+^ CD73^+^	● Converts ATP to generate adenosine (ADO) and dampens inflammation [32] ● Reduces pro-inflammatory cytokine production and maintains immune homeostasis	IL-10
TIM-1^+^	Both	CD19+ TIM-1+	● Suppresses Th1 and Th17 response ● Promotes immune tolerance	IL-10 TGF-β
PD-L1^+^	Both	CD19+ PD-L1+	● Suppresses T cell-mediated cytokines such as IFNγ and IL-21 in RA [38] ● Delays disease progression in EAE [23] ● Suppresses inflammation in autoimmune disease [23]	IL-10 TGF-β
Granzyme B^+^	Human	CD19^+^ CD5^+^ Granzyme B+	● Suppresses the effector CD4 T cell proliferation and cytokine production by granzyme B-mediated TCR cleavage	

## Breg-Mediated Immunosuppression

The immune system is charged with maintaining a delicate equilibrium: strong enough to eliminate pathogens and tumors yet restrained enough to avoid excessive inflammation or autoimmunity. Achieving this balance requires the coordinated activity of regulatory cell populations, with Bregs and Tregs working in tandem through complementary suppressive mechanisms. Whereas Tregs are well defined by their lineage marker FOXP3, the pathways governing Breg function remain less well characterized, though emerging evidence has established their critical role in restraining pathogenic T cell activity.

### Regulators of Bregs Function

**Fig. 2:**
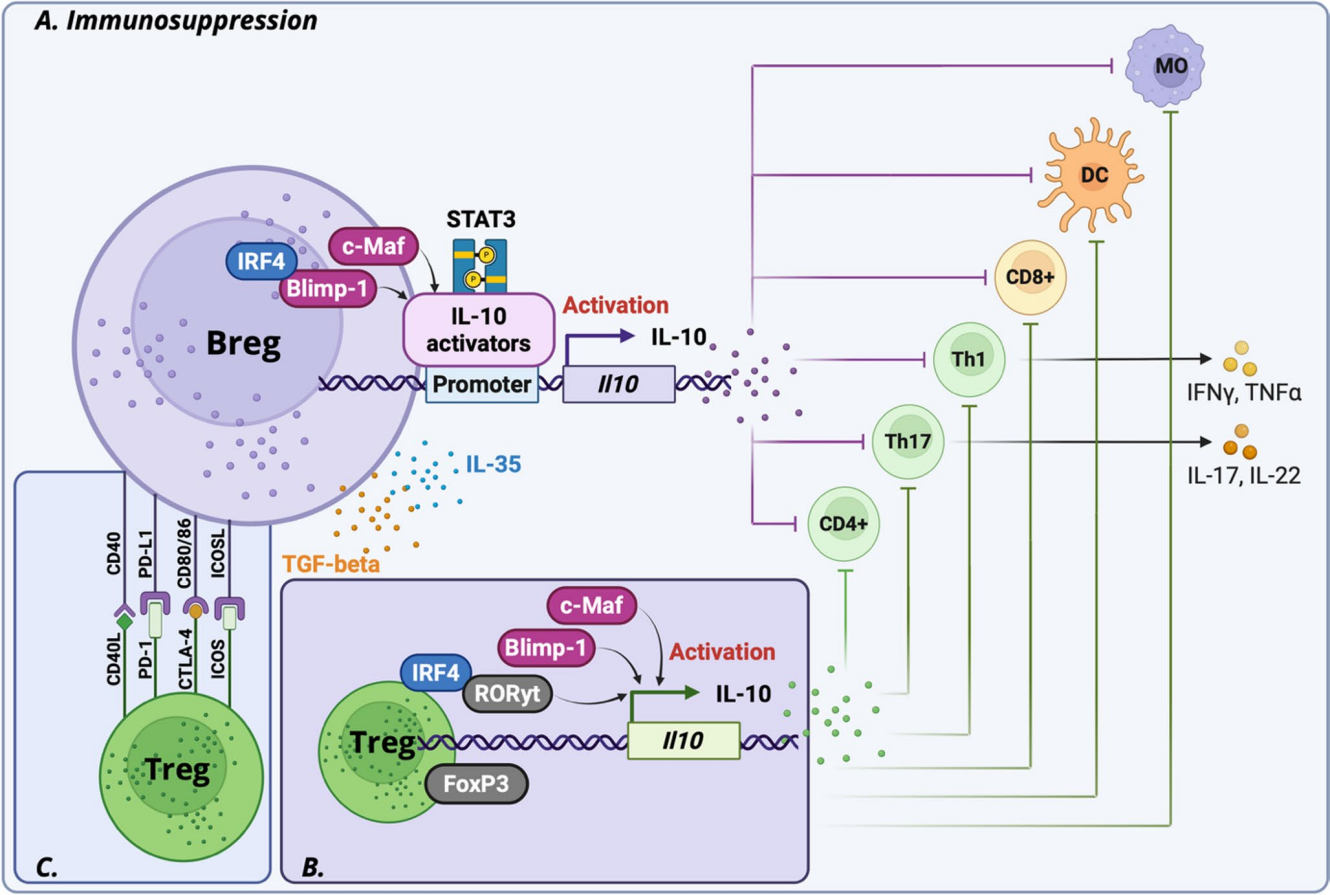
Proposed mode of Transcriptional crosstalk between regulatory B and T cells causes IL-10-mediated immunosuppression in both cell types. Possible interaction between immune regulatory receptors/ligands on Bregs and Tregs. 2 A. Blimp-1-STAT3 mediates IL-10 activation and immunosuppression in Bregs. 2B. Blimp-1-mediated transcriptional activation of IL-10 leads to immunosuppression within the immune environment of Tregs. 2C. Co-stimulatory and checkpoint interactions of Breg-Treg mediate immunosuppressive crosstalk

Bregs primarily exert suppression through cytokine secretion, with IL-10 as the dominant mediator, supported by IL-35 and TGF-ꞵ. These cytokines converge to inhibit Th1 and Th17 differentiation and proliferation, thereby dampening pro-inflammatory responses (Fig. 2). Among these mediators, IL-10 is considered the hallmark of Breg activity, and its expression is tightly controlled by transcriptional and epigenetic mechanisms tightly control its expression.

#### Blimp-1

A central regulator of IL-10 in Bregs is Blimp-1 (B lymphocyte-induced maturation protein 1, encoded by *Prdm1*). Blimp-1 orchestrates the differentiation of B cells into plasmablast-like Bregs that produce IL-10. The plasmablasts (CD138^+^) are a subset of the IL-10-producing Breg cells during autoimmune inflammation [43]. Additionally, Blimp-1 serves as a key transcriptional regulator of plasmablast differentiation [44]. We believe that the research gap concerning different Breg subtypes, especially regulatory B cells from various B cell subsets, remains to be fully explored. Functional studies have shown that IL-10-producing B10 cells (CD19^+^CD1d^high^CD5^+^) derived from Blimp-1 conditional knockout mice exhibit impaired suppression of naive CD4^+^ T cell proliferation, reflecting a loss of immunosuppressive capacity. Moreover, in *Prdm1* knockout mice, the transition of Bregs into terminal IL-10-producing plasmablasts is disrupted. Interestingly, Blimp-1 plays a dual role. In resting B10 cells, IL-10 expression is reduced by Blimp-1, but in activated B10 cells, Blimp-1 enhances IL-10 production, emphasizing its context-dependent activity [45]. Blimp-1 is a non-redundant transcriptional regulator of Breg immunosuppression, with its absence tipping the balance toward pro-inflammatory cytokine production and autoimmune pathology (Fig. 2).

#### STAT3

Blimp-1 functions synergistically with STAT3 to regulate the *Il10* promoter (Fig. 2). Upon immune activation, such as LPS stimulation, STAT3 binds directly to the *Il10* promoter, but efficient transcription requires cooperation with Blimp-1. The DNA-binding domain of Blimp-1 is essential for enabling the phosphorylated STAT3 (Tyr705) to fully activate IL-10 expression in B10 cells. This interdependence shows that STAT3 alone cannot drive *Il10* transcription, but with Blimp-1, it provides a potent transcriptional program for Breg-mediated suppression [45]. The importance of STAT3 in Bregs is further stressed by genetic studies. Within the literature, loss of STAT3 in B cells increases the severity of autoimmune uveitis, marked by reduced IL-10 and IL-35 levels [46].

Beyond IL-10, Blimp-1 also promotes the transcription of IL-35 by activating *Il12a* and *Ebi3* through the BATF/IRF4/IRF8 axis [47]. Among the IL-35 subunits, IL-12p35 has shown strong immunoregulatory potential, suppressing Th17 proliferation and expanding Breg populations [48]. In addition, STAT3 regulates the expression of the inhibitory receptor LAG-3 on Bregs. In STAT3-deficient mice, diminished LAG-3 expression compromises the ability of LAG-3⁺ Bregs to suppress Th17 expansion, further illustrating STAT3’s central role in sustaining Breg immunoregulatory function [46]. While STAT3 clearly regulates IL-10, whether it directly drives IL-35 expression in Bregs remains unresolved. Nonetheless, IL-35 is functionally significant, as it induces the expansion of IL-10⁺ Bregs and CD5⁺CD19⁺B220^lo^ Bregs, both of which suppress pathogenic T cell responses and ameliorate uveitis [8].

#### c-Maf

Another critical transcription factor, c-Maf, has also been shown to bind directly to the *Il10* promoter upon LPS stimulation, thereby augmenting IL-10 production in Bregs [49]. Upstream regulators further refine this transcriptional network. IRF4 activates *Prdm1* and induces Breg expansion, whereas IRF8 acts antagonistically, restraining this pathway (Fig. 2). The bifurcation of IRF4 and IRF8 activity thus determines the extent of Blimp-1 expression and downstream Breg differentiation [50].

#### HDAC

**Fig. 3:**
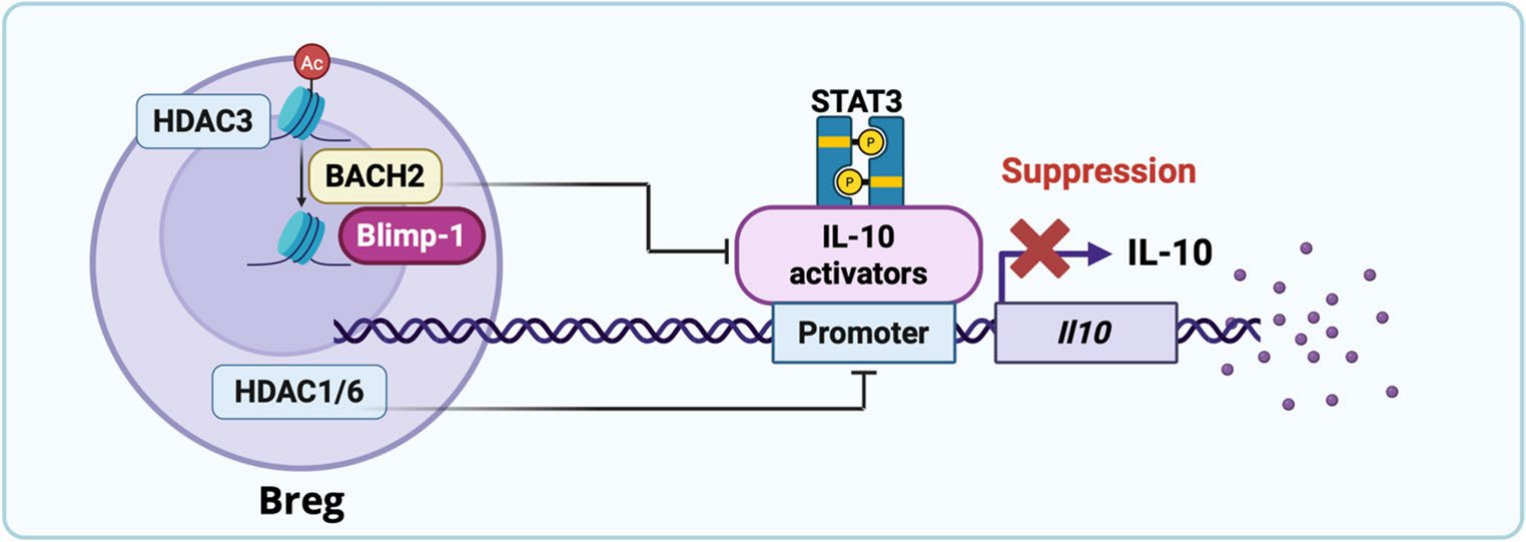
Proposed model of Epigenetic regulation of IL-10-mediated immunosuppression by histone deacetylases (HDACs) in Bregs

Epigenetic mechanisms superimpose additional layers of control. BACH2, a transcriptional repressor, directly suppresses Blimp-1 expression and limits plasma cell differentiation (Fig. 3). Human ChIP-seq data confirm that BACH2 binds the *Il10* promoter and represses IL-10 production in B cells [51, 52]. Mechanistically, BACH2 recruits HDAC3 to the *Prdm1* locus, driving deacetylation of H3K9 and silencing Blimp-1 transcription [53]. Although epigenetic programming of IL-10 competence is common, the recruitment of HDACs to chromatin upstream occurs in a context-specific manner. HDACs are epigenetic regulators of IL-10 expression in regulatory B cells. Inhibiting HDAC1 with Entinostat prevents it from binding to the nearby region of the IL-10 expression promoter in splenic B cells and IL-10-producing Bregs [54]. Additionally, inhibiting HDAC3 can promote the differentiation of plasma cells into CD138^hi^IL-10^+^IgM^+^ plasma cells [55], a distinct Breg subset, also regulated by epigenetic mechanisms, alongside the traditional Bregs (CD1d^hi^ CD5^+^ (B10)). However, the identity of Bregs is not determined solely by surface markers; instead, they are epigenetically programmed, which modifies chromatin accessibility and influences IL-10 production in Bregs [56]. BACH2 typically binds to the IL-10 gene promoter, and genetic removal of BACH2 binds to the IL-10 gene promoter, and knocking out BACH2 genetically results in increased IL-10 production in IL-10-producing regulatory B cells [52]. Additionally, the BACH2-HDAC3 co-repressor complex epigenetically binds to the Blimp-1 locus [53]. These findings collectively support our model of BACH2-dependent repression of IL-10 expression via the Blimp-1 regulator.

#### PD-L1

PD-L1-mediated suppression represents another key mechanism (Fig. 2). PD-L1 (CD274) expressed on Bregs engages PD-1 (CD279) on T cells, directly inhibiting TCR signaling and reducing CD8⁺ T cell proliferation. Clinical studies in rheumatoid arthritis have shown that CD19⁺PD-L1⁺ Bregs are significantly reduced in untreated patients, while treatment restores their frequency and enhances T-cell suppression [57]. Myeloid-derived suppressor cells (MDSCs) also contribute to this pathway: MDSCs promote PD-L1 expression on Bregs, thereby enhancing their capacity to suppress CD8⁺ T cell proliferation. Notably, when PD-L1⁻ B cells are substituted in co-culture systems, this suppressive effect is lost, confirming the dependence of Breg-mediated suppression on PD-L1 [58].

Breg-mediated immunosuppression is a multilayered program integrating cytokine production, transcriptional and epigenetic regulation, and receptor-mediated signaling. IL-10 remains the central axis of this program, but its stabilization relies on the interactions of Blimp-1, STAT3, c-Maf, IRF4, and BACH2, as well as epigenetic modifiers such as HDACs (Figs. 2 and 3) Parallel pathways, including CD40-CD40L interactions and PD-L1 checkpoint signaling, expand the suppressive repertoire of Bregs (Fig. 2). Unlike Tregs, whose suppressive program is well established, Bregs represent a more fluid and plastic population, with their identity and function tightly controlled by the convergence of transcriptional networks and environmental cues.

Regulatory B cells enhance CD4 + regulatory T cell responses by secreting IL-10, primarily via an IL-10-dependent pathway that interacts directly with IL-10 receptors on CD4 T cells. Breg-derived IL-10 activates the STAT3 pathway, which promotes FOXP3 expression, turning T cells into more functional regulatory T cells and suppressing pro-inflammatory differentiation of Th17 and Th1 cells. Additionally, regulatory B cells produce TGF-beta, inducing FOXP3 transcription through SMAD2/3 in CD4 T cells. TGF-beta stimulates FOXP3 transcription and inhibits Th1/Th2 effector CD4^+^ T cell differentiation. The CTLA-4 on Tregs interacts with CD80 or CD86 on Bregs, enhancing Treg-mediated suppression. Moreover, Bregs’ expression of PD-L1 can bind to PD-1 on T cells, reducing PI3K/Akt/mTOR signaling and RORyt expression while increasing FoxP3 levels in Tregs. Overall, the epigenetic stabilization of the IL-10 locus in regulatory Bregs and the FOXP3 locus in Tregs underpins their crosstalk, although more research is necessary to understand the epigenetic profiles of Breg-Treg interactions.

### Bregs in Autoimmune Disease

**Fig. 4:**
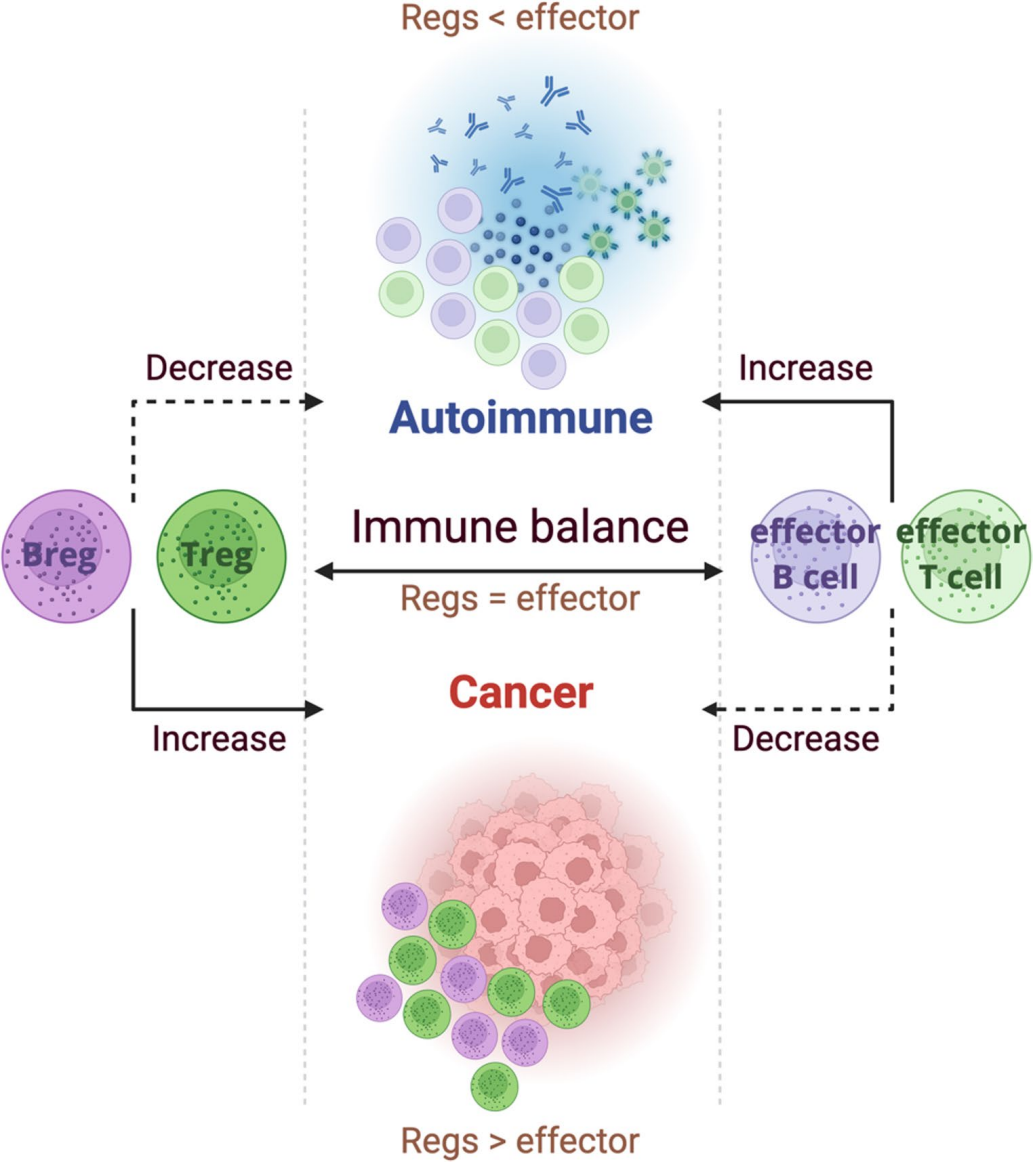
The dual immunoregulatory functions of Bregs and Tregs in cancer and autoimmune disease. Both autoimmune diseases and cancer illustrate opposing mechanisms of immune regulation. Autoimmune diseases involve a breakdown of immune tolerance, often linked to reduced numbers of regulatory B cells and T cells. Conversely, cancer creates a highly immunosuppressive tumor microenvironment characterized by increased Bregs and Tregs, which inhibit the responses of effector T and B cells. Unbalance of this “immune balance” could lead to autoimmunity (Regs < Effectors) or cancer development (Regs > Effectors). Regs: Regulatory cells, effector: Effector cells

The immunosuppressive axis of the immune system is sustained through the coordinated activity of Tregs and Bregs, both of which serve to limit inflammation-associated tissue damage and preserve immune homeostasis (Fig. 4). By reinforcing self-tolerance, these regulatory populations counteract autoimmune pathology and restrain aberrant immune activation. This tolerance is achieved through multiple mechanisms, including the elimination of autoreactive lymphocytes, the secretion of suppressive cytokines such as IL-10, IL-35, and TGF-β, and the modulation of antigen-presenting cell (APC) activity. While Tregs primarily exert their effects by directly suppressing effector T cell responses, Bregs function as versatile immune modulators, shaping both innate and adaptive pathways to maintain balance within the immune system.

#### Multiple Sclerosis (MS)

Multiple sclerosis (MS) is a chronic autoimmune disorder of the central nervous system (CNS), defined by immune-mediated demyelination and progressive neurodegeneration. Disease pathology is orchestrated by autoreactive T helper subsets, particularly Th1 and Th17 cells, which establish an inflammatory cascade that drives both tissue injury and clinical progression [59].

Regulatory B cells play a central role in mitigating this pathology. B cell-derived IL-10 has been shown to reduce the severity of Th1-driven experimental autoimmune encephalomyelitis (EAE), the murine model of MS^53^. Within this context, CD40 signaling is required to induce IL-10 production in Bregs upon antigen encounter, demonstrating the importance of co-stimulatory pathways in shaping the suppressive phenotype of B cells [60]. Conversely, loss of CD40-CD40L signaling impairs Breg function in models of EAE and collagen-induced arthritis, underscoring its importance in autoimmune regulation [61]. The timing of regulatory activity also appears critical: B10 cells (CD1d^hi^CD5^+^IL-10^+^) exert their strongest anti-inflammatory effects during the early stages of EAE progression, whereas FOXP3^+^ Tregs predominantly regulate later phases of disease [62, 63].

In MS patients, however, these regulatory mechanisms are compromised. Although frequencies of CD4⁺CD25^hi^ Tregs are not significantly altered compared with healthy controls, their suppressive capacity is diminished. Tregs isolated from MS patients fail to efficiently suppress effector T cells stimulated with recombinant myelin oligodendrocyte glycoprotein (MOG), demonstrating functional defects in antigen-specific tolerance [64]. Importantly, this impairment is observed across both relapsing and remission phases of disease, suggesting that Treg dysfunction is a persistent feature of MS rather than a state-specific defect. Consistent with this, Th17-mediated inflammation correlates with disease severity, and an inverse relationship between Tregs and Th17 cells has been reported [65].

Bregs, by contrast, show a more direct association with disease activity. Frequencies of IL-10⁺ Bregs positively correlate with MS severity [66]. However, their functional capacity appears compromised: in relapsing-remitting MS (RRMS), the differentiation of B cells into CD19⁺CD24^hi^CD38^hi^ transitional Bregs is impaired, weakening their regulatory contribution. Notably, this dysfunction can be at least partially restored by treatment with the immunomodulatory peptide thymosin-α1 (T-α1), which enhances IL-10 production and re-establishes suppressive activity [67]. The push-and-pull between Tregs and Bregs in MS leads to deficiencies in both populations, which contribute to a permissive environment for Th1/Th17-driven neuroinflammation.

#### Systemic Lupus Erythematosus

The contribution of Bregs to the pathogenesis of systemic lupus erythematosus (SLE) remains incompletely defined, yet accumulating evidence points to both quantitative and functional defects in this population. Transitional Bregs (CD19⁺CD24^hi^CD38^hi^), typically a major source of IL-10, are reduced in frequency and display impaired IL-10 production in SLE patients [24]. These defects are particularly pronounced in individuals with lupus nephritis, where compromised Breg function correlates with loss of immunosuppressive control and enhanced disease activity [68]. Deficiencies in regulatory T cells add to this dysregulation. Circulating CD4⁺ Tregs are significantly reduced in SLE patients compared to both rheumatoid arthritis (RA) cohorts and healthy controls [69, 70]. Moreover, residual Tregs in SLE have impaired suppressive function, suggesting that failures across multiple regulatory cell compartments synergize to promote immune dysregulation.

The role of IL-10⁺ Bregs in lupus is also context-dependent. In lupus-prone mice, marginal zone Bregs appear early in disease, whereas plasmablasts and plasma cells dominate the IL-10-producing Breg compartment during active disease flares. Despite their expansion, these subsets exhibit functional impairment, as evidenced by elevated inflammatory gene signatures in lupus-prone MRL/lpr mice. This suggests that while Breg-like populations emerge during active disease, their regulatory function is blunted, allowing unchecked inflammation to persist [71]. Regulatory dysfunction in SLE extends beyond T cells to include both quantitative and qualitative defects in Bregs. Rather than restraining autoimmunity, impaired IL-10 production and context-dependent dysfunction may transform Bregs into insufficient or even maladaptive regulators of disease progression.

#### Inflammatory Bowel Disease (IBD)

Regulatory B cells also contribute to the control of intestinal inflammation, with B10 cells (CD1d^hi^CD5⁺IL-10⁺) emerging as a central subset. In dextran sulfate sodium (DSS)-induced models of colitis, B10 cells ameliorate intestinal injury, paralleling the protective effects observed in patients with ulcerative colitis [41]. Within the mesenteric lymph nodes (MLN), B cells demonstrate elevated CD1d expression, enhancing IL-10 production and reinforcing local immunoregulation. Interestingly, while enlarged MLNs have been reported in TCRα^−/−^, IL-2^−/−^, and IL-10^−/−^ mice, CD1d upregulation was specifically observed in TCRα^−/−^ and IL-deficient strains, suggesting that CD1d-driven Breg responses are engaged during inflammatory conditions [7]. Genetic models further reinforce the importance of B cells in intestinal homeostasis. TCRα^−/−^ × Igµ^−/−^ mice develop more severe spontaneous colitis than TCRα^−/−^ mice alone, indicating that B cells exert a suppressive role rather than serving as initiators of intestinal inflammation [72]. Similarly, CD1d-deficient mice spontaneously develop intestinal inflammation, reinforcing the idea that Bregs, and particularly IL-10-producing B10 cells, are required to prevent uncontrolled mucosal immune activation [7, 72]. CD1d is a molecule that bridges Bregs and iNKT cells (invariant Natural Killer T cells) and helps stabilize the IL-10-producing Breg function. Its main role is to present lipid antigens. CD1d-mediated engagement of iNKT cells is necessary for maintaining and expanding functional Bregs, but CD1d alone does not support the intrinsic IL-10 transcription within Bregs.

Other Breg subsets also play protective roles in the gut. IL-33-responsive Bregs (Breg^IL−33^) suppress the onset of spontaneous colitis in an IL-10-dependent manner, suggesting a link between epithelial alarmin signaling and the activation of suppressive B-cell networks that preserve mucosal immune tolerance [73]. In humans, frequencies of CD24^hi^CD38^hi^ and CD5⁺ Bregs are significantly decreased in both peripheral blood and intestinal tissues of ulcerative colitis patients compared with healthy controls [74]. The reduction in this subset correlates inversely with disease severity, supporting a protective role for transitional-like Bregs in maintaining mucosal balance. Conversely, expansion of CD95⁺ Bregs has been associated with functional impairment, reflecting a skewing of Breg subsets toward a non-suppressive phenotype in inflammatory conditions [75]. Bregs are essential for maintaining intestinal immune homeostasis. Their impairment, whether by numerical reduction, altered signaling, or functional exhaustion, facilitates chronic inflammation and contributes to the pathogenesis of inflammatory bowel disease.

#### Rheumatoid Arthritis (RA)

Rheumatoid arthritis (RA) is a chronic autoimmune disease characterized by persistent joint inflammation, synovial hyperplasia, and progressive cartilage and bone destruction. Both innate and adaptive immune pathways contribute to disease pathology, with Th1- and Th17-driven responses playing a central role. Regulatory B cells, particularly IL-10-producing subsets, have been shown to counterbalance this inflammatory axis. Evidence from collagen-induced arthritis (CIA), a well-established murine model of RA, showcases that B cell-derived IL-10 is critical for controlling disease progression. In chimeric mice lacking IL-10^−/−^ B cells, CIA severity is markedly increased, accompanied by amplified Th1 and Th17 responses, accentuating the non-redundant role of Bregs in restraining joint inflammation [76].

In human RA, several Breg subsets are numerically and functionally impaired. CD19⁺CD27⁺IL-10⁺ memory Bregs are reduced in frequency compared with healthy individuals, and their diminished regulatory capacity is reflected in a failure to suppress CD4⁺ T cell-derived IFN-γ production [77]. Similarly, the IL-10-producing B10 population, typically enriched within the transitional CD19^+^CD24^hi^CD27^+^ compartment, is significantly decreased in the peripheral blood of RA patients [78]. Checkpoint-regulated subsets are also affected. PD-L1⁺ Bregs, which normally suppress effector cytokine production, are reduced in RA and possess functional impairment. In particular, PD-L1⁺ B cells from RA patients fail to suppress IFN-γ and IL-21 secretion, further expanding upon defects in Breg-mediated regulation [38].

Impaired frequency and function of multiple Breg subsets, transitional, memory, and checkpoint-linked, contribute to the unchecked pro-inflammatory environment in RA. Loss of Breg-mediated regulation thus permits persistent Th1/Th17 activity, exacerbating joint inflammation and autoimmunity.

#### Type 1 Diabetes

Type 1 diabetes (T1D) is an autoimmune disease characterized by the destruction of pancreatic β cells, leading to insulin deficiency and lifelong dependence on exogenous insulin. Dysregulated immune tolerance plays a central role in disease onset, and emerging evidence implicates Breg deficiency in the failure to restrain autoreactive T cells. In patients with T1D, circulating transitional Bregs (CD24^hi^CD38^hi^) are significantly reduced in frequency compared with healthy controls [79]. Additional reductions in TIM-1⁺ Bregs and IL-10-producing Bregs have also been observed, suggesting a global defect across multiple Breg subsets [79].

Similarly, the frequency of IL-35⁺ Bregs, defined as CD19⁺CD24⁺CD40⁺CD38⁺ in humans, is diminished in peripheral blood mononuclear cells (PBMCs) of T1D patients. In the non-obese diabetic (NOD) mouse model, IL-35⁺ Bregs (CD19⁺CD1d⁺CD5⁺) are reduced in both the spleen and pancreatic draining lymph nodes, displaying a parallel impairment in murine autoimmune diabetes [80]. Mechanistic studies suggest that antigen-matured Bregs sustain tolerance by suppressing autoreactive T cells and supporting the survival and function of pancreatic β cells. In this way, Bregs may act as suppressors of inflammation and facilitators of β-cell preservation, offering a potential therapeutic axis for restoring endogenous insulin production in T1D [81].

### Bregs in Cancer

In contrast to autoimmune diseases, where deficiencies in regulatory populations exacerbate inflammation, Tregs and Bregs in cancer act to create an immunosuppressive tumor microenvironment (TME) that favors immune evasion and tumor progression (Fig. 4). Within the TME, both regulatory cell types suppress antitumor immunity by limiting effector T cell activation and cytokine production, thereby enabling tumor cells to persist and proliferate.

The heterogeneity of regulatory B cell subsets complicates their characterization in cancer, but functional parallels with Tregs are evident. Both Tregs and Bregs converge on the production of immunosuppressive cytokines, including IL-10, IL-35, and TGF-β, and engage checkpoint pathways such as PD-1/PD-L1 to attenuate cytotoxic T lymphocyte activity. This overlap highlights a common immunoregulatory program, adapted in cancer to protect malignant rather than host tissues.

#### Breast Cancer

In breast cancer, regulatory B cells play a pivotal role in shaping a tolerogenic tumor microenvironment that facilitates metastasis and immune evasion. Mouse models using the 4T1 mammary carcinoma cell line, a highly metastatic model that closely mimics the biology of human triple-negative breast cancer, have revealed the emergence of a specialized subset termed tumor-evoked Bregs (tBregs). Unlike conventional IL-10-producing Bregs, tBregs exert their immunosuppressive function primarily through TGF-β, which reprograms naïve CD4⁺ T cells into FOXP3⁺ Tregs. This process expands the immunosuppressive compartment and promotes tumor progression and metastasis [82].

Beyond their role in Treg induction, Bregs within the breast cancer microenvironment actively secrete anti-inflammatory cytokines and suppress effector T cell responses, thereby reinforcing a state of immune tolerance that favors tumor outgrowth. Mechanistically, the generation of tBregs has been linked to lipid metabolism [83]. Specifically, the 5-lipoxygenase (5-LO)/FLAP/LT pathway and PPAR-α signaling have been implicated as critical regulators of Breg differentiation in breast cancer. Inhibition of these pathways disrupts tBreg development and breaks the Breg-driven immunosuppressive loop, punctuating their importance in sustaining tumor-promoting regulation [84].

Tregs likewise maintain a dominant immunosuppressive role in breast cancer, particularly within tumor-draining lymph nodes (TDLN). High accumulation and functional activation of FOXP3⁺ Tregs in these sites correlate with poor prognosis, demonstrating the cooperative nature of Breg- and Treg-mediated suppression in metastatic breast cancer [85]. In breast cancer, Bregs and Tregs do not act in isolation and form a synergistic immunoregulatory axis. Through both cytokine-mediated mechanisms and metabolic reprogramming, this axis establishes an immune-privileged niche that accelerates tumor progression and metastasis.

#### Colorectal Cancer

In colorectal cancer (CRC), regulatory B cells have been identified among tumor-infiltrating lymphocytes, where they contribute to the establishment of a tolerogenic microenvironment. A distinct subset of IL-10⁺ plasmablast-like Bregs, characterized by CD19^lo^CD27^hi^ expression, has been observed in resected CRC tissues. When co-cultured with peripheral blood CD3⁺ T cells, these CD19^lo^CD27^hi^ plasmablasts suppressed effector function, leading to reduced secretion of pro-inflammatory cytokines such as IFN-γ and TNF-α, in contrast to CD19^hi^CD27⁻ naïve B cells. These IL-10⁺ plasmablasts also expressed TIM-1 and IgG, but patients with colorectal tumors exhibited decreased frequencies of CD38⁺CD24⁺ transitional Bregs in peripheral blood, suggesting a systemic deficiency in immunoregulatory populations [86].

Additional insights into colitis-associated cancer (CAC), a subtype of CRC linked to chronic intestinal inflammation, further the relationship between Bregs, microbiota, and tumor progression. IgA⁺ plasma cells regulate CAC by shaping a tumor-protective microbial environment that dampens bacterial-driven inflammation. Concurrently, IL-10-producing Bregs restrain pathogenic Th1 and Th17 responses during chronic colitis, reducing the inflammatory signals that drive malignant transformation [87].

In CRC, Bregs operate both locally within the tumor and systemically to limit pro-inflammatory immunity. By curbing effector T cell responses and interacting with microbiota-dependent pathways, Bregs contribute to an immune landscape that paradoxically protects against excessive inflammation but also fosters tumor persistence and progression.

#### Hepatocellular Carcinoma (HCC)

In hepatocellular carcinoma (HCC), Bregs play a prominent role in dampening antitumor immunity and shaping an immunosuppressive tumor microenvironment. Experimental strategies have sought to disrupt these pathways. For example, an engineered CD19scFv-IL-10R fusion protein, designed to bind both CD19 and IL-10, was tested in murine models of HCC [88]. Treatment with this construct suppressed B10 Bregs and Tregs while preserving Th1 responses, leading to accumulation of IFN-γ-producing CD4⁺ T cells and cytotoxic CD8⁺ T cells that directly targeted tumor cells. The result was significantly reduced tumor growth, demonstrating the therapeutic potential of selectively interrupting Breg/Treg-mediated suppression [88].

Clinically, specific Breg subsets correlate with disease progression. A PD-1^hi^ Breg population, characterized by CD5^hi^CD24^hi^CD27^+^CD38^dim^ expression, has been linked to poor outcomes in HCC. These cells suppress T cell-mediated antitumor responses and thereby facilitate tumor persistence [89]. In addition, TIM-1⁺ Bregs, already implicated in other tumor settings, are enriched in HCC. TIM-1⁺ B cells are associated with reduced overall survival and worse prognosis compared with TIM-1⁻ counterparts, suggesting that TIM-1 may serve both as a functional marker of immunosuppressive Bregs and a prognostic biomarker in HCC [90]. In HCC, Bregs suppress cytotoxic immune surveillance through both checkpoint- and cytokine-driven mechanisms. Targeting these pathways, either by disrupting IL-10 signaling or by inhibiting PD-1⁺ and TIM-1⁺ Breg subsets, may represent a viable therapeutic strategy to restore effective antitumor immunity in liver cancer.

#### Melanoma

In melanoma, Bregs contribute directly to tumor progression by suppressing cytotoxic immunity and fostering a pro-tumorigenic microenvironment. Experimental studies using the B16F10 melanoma model demonstrated that B1a Bregs (CD1d^int^CD5⁺), which secrete IL-10, accelerate tumor growth when adoptively transferred into recipient mice. This effect was associated with impaired production of Th1 cytokines and diminished effector function in tumor-infiltrating CD8⁺ T cells, implicating IL-10 as a central mediator of Breg-driven suppression [17].

Beyond cytokine secretion, Bregs in melanoma also exploit signaling pathways that enhance tumor survival. CD5⁺ B cells have been shown to directly bind IL-6, initiating STAT3 activation, which in turn promotes tumor growth through transcriptional programs that support both immune suppression and tumor proliferation [91]. Clinical observations further reinforce the link between Bregs and melanoma progression. Circulating PD-L1⁺ Bregs are elevated in patients with advanced-stage melanoma (stages III-IV), with the highest frequencies observed in those with bone metastases compared to patients with primary tumors. This finding stresses the role of PD-1/PD-L1-mediated suppression in facilitating systemic immune escape during metastatic disease [37].

In addition, a distinct subset of IgG4⁺CD49b⁺CD73⁺ Bregs has been identified in melanoma, which promotes angiogenesis and tumor-associated vasculature. This population operates at the interface of immunoregulation and tumor remodeling, further broadening the spectrum of Breg-mediated mechanisms that sustain melanoma progression [92]. Bregs act as multifaceted contributors to melanoma progression, acting through IL-10-mediated suppression of effector T cells, IL-6/STAT3-driven pro-tumor signaling, checkpoint regulation, and the promotion of angiogenesis.

#### Pancreatic Cancer

In pancreatic cancer, regulatory B cells contribute to tumor initiation and progression by reinforcing an immunosuppressive environment during both early neoplasia and established disease. In vivo models of pancreatic tumorigenesis have identified an enrichment of CD19⁺CD1d^hi^CD5⁺IL-35⁺ B cells within early neoplastic lesions. These Bregs promote tumor growth, in part by driving immune tolerance, showcasing their role at the earliest stages of malignant transformation [93].

During chronic inflammation associated with pancreatic cancer, IL-35 expression is further upregulated. Mechanistically, the IL-35 subunit p35 (Il12a) is predominantly active in Bregs, whereas the Ebi3 subunit is strongly induced in CD4⁺ T cells, amplifying IL-35 signaling and contributing to pancreatic neoplasia [94]. Importantly, IL-35⁺ Bregs activate STAT3 through phosphorylation, providing a direct link between Breg-derived cytokines and tumor-promoting signaling cascades.

In pancreatic ductal adenocarcinoma (PDA), an increased frequency of CD19⁺CD24^hi^CD38^hi^ Bregs has been observed in peripheral blood, where their accumulation correlates with impaired CD8⁺ T cell effector function compared to healthy individuals. Bregs contribute to systemic immune suppression in addition to their local effects within the tumor microenvironment. Notably, blockade of IL-35 signaling has been proposed as a therapeutic strategy to restore CD8⁺ T cell infiltration and overcome resistance to immunotherapy in PDA [95].

IL-35⁺ Bregs act as central mediators of pancreatic cancer progression. By activating STAT3 signaling and suppressing cytotoxic lymphocyte activity, they reinforce a microenvironment conducive to immune evasion and therapeutic resistance. Targeting this axis may open new avenues for effective immunotherapy in pancreatic malignancy.

#### Non-small Cell Lung Cancer (NSCLC)

In non-small cell lung cancer (NSCLC), regulatory B cells act through both systemic and tumor-localized alterations that contribute to immunosuppression. Peripheral blood from NSCLC patients shows an enrichment of transitional Bregs (CD24^hi^CD38^hi^), while within the tumor microenvironment, Bregs are more frequently characterized by IL-10⁺CD138⁺ phenotypes, often co-expressing PD-L1. These subsets collectively reinforce a suppressive environment that dampens antitumor immune responses [29]. In addition to conventional subsets, a distinct Breg population has been described in NSCLC patient blood, defined by CD38⁺CD138^dim^Ki-67⁺IL-10^dim^ and VISTA⁺ expression [29, 96]. This subset exhibits enhanced metabolic fitness, engaging glycolysis, the tricarboxylic acid (TCA) cycle, and fatty acid oxidation, suggesting an adaptation that promotes survival and persistence within metabolically demanding tumor environments.

Importantly, these VISTA⁺ Bregs may colocalize with P-selectin glycoprotein ligand-1 (PSGL-1) on T cells within tertiary lymphoid structures (TLS). Functional studies revealed that the VISTA-PSGL-1 interaction correlates positively with NSCLC recurrence, pointing to a mechanism by which Bregs exploit checkpoint pathways to reinforce tumor immune evasion [96]. In NLSCLC, Bregs function as metabolic and checkpoint regulators, operating through IL-10, PD-L1, and VISTA-dependent pathways. Their capacity to adapt metabolically while engaging in direct immunosuppressive signaling highlights their dual role in shaping the TME and driving recurrence after therapy.

#### Glioblastoma (GBM)

Glioblastoma (GBM), the most aggressive form of primary brain tumor, is marked by profound immunosuppression within its microenvironment. Bregs contribute significantly to this immune dysfunction. A subset of PD-L1⁺ Bregs has been identified in GBM, where their suppressive function is maintained through interactions with myeloid-derived suppressor cells (MDSCs). This signaling axis reinforces T cell suppression and accelerates GBM progression, displaying a cooperative network of regulatory cells within the tumor [97].

In addition to checkpoint regulation, cytokine-driven mechanisms also promote Breg function in GBM. Elevated TGF-β levels in the GBM microenvironment, primarily activated through αVβ8 integrin signaling, foster the induction of immunosuppressive Bregs. Notably, combined therapeutic blockade of PD-1 and αVβ8 integrin has been shown to restore B and T cell proliferation and rescue plasmablast differentiation, suggesting that dual targeting of checkpoint and integrin pathways may disrupt Breg-mediated suppression and enhance antitumor immunity [98].

#### Prostate Cancer

Prostate cancer is one of the most prevalent malignancies in men worldwide and is associated with a strong immunosuppressive component within its tumor microenvironment. Regulatory B cells are enriched in prostate cancer patients, with increased frequencies of CD19⁺IL-10⁺ B cells reported compared with individuals with benign prostatic hyperplasia (BPH). This expansion of IL-10-producing Bregs suggests an enhanced immunoregulatory network that may contribute to tumor persistence and progression [99].

In parallel, regulatory T cells are also elevated in prostate cancer. Tumor-infiltrating lymphocytes (TILs) isolated from prostate tumors show a significant increase in CD4⁺CD25⁺ Tregs, further strengthening the suppressive microenvironment. The accumulation of these Tregs has been linked with reduced effector T cell responses and poor immune surveillance [100]. The combined presence of Bregs and Tregs within the TME indicates potential therapeutic targets aimed at disrupting IL-10 production or Treg expansion to restore antitumor immunity.

### Clinical Relevance of Regulatory B Cells

Clinical evidence suggests that regulatory B cells (Bregs) are involved in understanding disease progression and patient outcomes, depending on the context. Based on the previous studies, the cord blood is enriched with CD19^+^CD24^hi^CD38^hi^ regulatory B cells, also in peripheral blood [11]. The infiltrating Bregs (CD19^+^CD20^+^CD24^hi^CD38^hi^) in tumor-draining lymph nodes (TDLNs) and non-tumor-draining lymph nodes (n-TDLNs) vary when compared to patients without lymph node metastases [101]. These patients are from oral squamous cell carcinoma. Additionally, IL-10 production by B cells in HNSCC patients’ lymph nodes is significantly higher in TDLNs than in nTDLNs (25.2% vs. 7.27%). Furthermore, elevated levels of regulatory B cells (IL-10/CD19) in tongue squamous cell carcinoma (TSCC) are associated with poor patient survival [102]. These Bregs encourage the transformation of resting CD4^+^ T cells into regulatory T cells (CD4^+^CD25^+^). An increased proportion of Bregs and Tregs in the serum of cervical cancer patients correlates with poorer survival outcomes [103]. Additionally, IL-10-producing Bregs were elevated in the peripheral blood mononuclear cells (PBMCs) of gastric tumor patients. Functional studies indicated that immunosuppression, as evidenced by inhibition of CD4^+^ T helper cell proliferation and CD19^+^ CD24^hi^CD38^hi^ Breg proliferation, was positively associated with CD4^+^FoxP3^+^ Tregs.

### Conclusion

Across diverse tumor types, including breast, colorectal, hepatocellular, melanoma, pancreatic, lung, glioblastoma, and prostate cancers, regulatory B cells emerge as key architects of the immunosuppressive tumor microenvironment. Unlike in autoimmunity, where Breg deficiency promotes disease, in cancer, their expansion or functional reprogramming reinforces tumor immune evasion (Fig. 4). These cells suppress effector T cell activity through multiple converging mechanisms, including secretion of IL-10 and IL-35, checkpoint engagement via PD-1/PD-L1 or VISTA, metabolic rewiring, and promotion of angiogenesis and stromal remodeling. Importantly, Bregs often act in synergy with Tregs, establishing a multilayered suppressive axis that blunts antitumor immunity and undermines therapeutic responses.

The tumor-promoting functions of Bregs are context-dependent, shaped by tumor type, microenvironmental cues, and metabolic states. For example, tBregs in breast cancer reprogram naïve CD4⁺ T cells into FOXP3⁺ Tregs, PD-L1⁺ Bregs are enriched in advanced melanoma and HCC, and IL-35⁺ Bregs drive pancreatic neoplasia via STAT3 activation. Emerging evidence from NSCLC and GBM further explores how Bregs exploit metabolic and integrin-dependent pathways to sustain suppression. These observations position Bregs as markers of immune evasion and promising therapeutic targets, where selective disruption of their suppressive circuits could restore antitumor immunity without abolishing their physiological role in maintaining tolerance.

### Final Discussion and Concluding Remarks

The expanding appreciation of Bregs as regulators of immune tolerance places them alongside FOXP3⁺ Tregs as central players in shaping immune outcomes across disease contexts. In autoimmunity, deficiencies in Bregs, whether numerical, phenotypic, or functional, undermine tolerance and permit pathogenic Th1 and Th17 responses to dominate. This is evident in multiple sclerosis, systemic lupus erythematosus, inflammatory bowel disease, rheumatoid arthritis, and type 1 diabetes, where impaired IL-10 production or loss of regulatory subsets directly correlates with disease severity and progression. By contrast, in cancer, Bregs are often co-opted or expanded to reinforce immunosuppressive programs, enabling tumors to escape immune surveillance and resist therapy.

Epigenetic regulation emerges as a unifying thread linking Breg function across these settings. Histone deacetylases (HDACs), transcriptional repressors such as BACH2, and transcription factors including Blimp-1, STAT3, and c-Maf converge at the IL-10 locus to regulate Breg identity and function (Figs. 2 and 3). The stability of regulatory B cells reflects their sustained regulatory function, primarily through their continued production of suppressive cytokines, mainly IL-10. Through this immunosuppressive function of Bregs can dampen the T cell response [2]. In addition to IL-10-producing B cell subsets, identified IL-35-producing B cells, particularly CD138^+^ plasma cells, are identified during EAE (encephalomyelitis), which are essential for sustaining their suppressive function. The plasticity of Bregs illustrates how B cells can adapt their immunosuppressive functions, which are vital for preserving a protective immune response and minimizing tissue damage in autoimmune disorders and allergies. In cancer, this adaptability may hinder effective anti-tumor immunity. Regulatory B cells are not strictly tied to a specific lineage; instead, they are defined by their function and can originate from various B cell lineages, including marginal zone-like B cells, plasma cells, and transitional B cells. Their regulatory role depends on signals and can be reversed through the B cell receptor, CD40 co-stimulatory signals, or Toll-like receptor activation. Overall, this indicates that Breg-mediated immune responses are context-sensitive and flexible, supporting immune tolerance during inflammatory states. This epigenetic plasticity highlights the potential for therapeutic interventions that fine-tune Breg activity, either restoring their suppressive function to temper autoimmunity or restraining their expansion in cancer to enhance immune surveillance.

The duality of Breg biology, protective in autoimmunity yet detrimental in cancer, underscores the need for precision strategies. Rather than indiscriminately targeting Bregs, future approaches must account for disease context, subset heterogeneity, and epigenetic states. Therapies aimed at enhancing Breg function may offer benefit in autoimmunity, as suggested by preclinical models where IL-10 induction restores tolerance. Conversely, strategies that block Breg-derived IL-10, IL-35, PD-L1, or VISTA signaling, or disrupt their metabolic fitness, may provide powerful adjuvants to checkpoint blockade and other immunotherapies in cancer.

Looking forward, single-cell transcriptomics, spatial profiling, and epigenomic mapping will be critical to resolving the heterogeneity of Bregs across tissues and disease states. Such efforts will refine our understanding of how Bregs integrate into broader immunoregulatory networks, particularly their dynamic relationship with Tregs, myeloid suppressor cells, and stromal elements. Ultimately, delineating the molecular checkpoints that govern Breg plasticity may unlock novel therapeutic opportunities to recalibrate the immune system, either to restore tolerance in autoimmunity or to break tolerance in cancer.

In summary, Bregs represent a double-edged sword of immunoregulation. Their ability to modulate inflammation, either by safeguarding tissue integrity or by shielding tumors, reflects their central role in immune homeostasis. Harnessing or restraining Bregs with precision will define the next frontier of therapeutic immunomodulation, offering new possibilities for treating autoimmune disease, chronic inflammation, and cancer. 

## Data Availability

No datasets were generated or analysed during the current study.
